# Continuous Real-Time Neuropsychological Testing during Resection Phase in Left and Right Prefrontal Brain Tumors

**DOI:** 10.3390/curroncol30020156

**Published:** 2023-02-06

**Authors:** Barbara Tomasino, Ilaria Guarracino, Tamara Ius, Miran Skrap

**Affiliations:** 1 Scientific Institute IRCCS “Eugenio Medea”, Polo FVG, Pasian di Prato, 33037 Udine, Italy; 2 Unità Operativa di Neurochirurgia, Azienda Sanitaria Universitaria del Friuli Centrale, 33100 Udine, Italy

**Keywords:** neuropsychology, glioma, executive functions, frontal lobe, awake surgery

## Abstract

Background: Executive functions are multi-component and are based on large-scale brain networks. For patients undergoing brain surgery in the prefrontal cortex, resection in the anterior prefrontal sites is assisted by continuous monitoring of their performance on several tasks measuring components of executive functions. In this study, we did not test patients during direct cortical stimulation, but during resection itself. We chose tests routinely used to assess executive functions and included them in a protocol for left (LH) and right (RH) hemisphere prefrontal resections. This protocol is meant to be used during real-time neuropsychological testing (RTNT)—an already established monitoring technique. Methods: We retrospectively reviewed a consecutive series of 29 adult patients with glioma in the superior and middle frontal areas who performed the RTNT sequence throughout the resection phase. The testing protocol comprised 10 tests for LH frontal resections and 9 tests for RH frontal resections. Results: RH patients showed a median performance on RTNT with significantly lower scores for visuo-spatial attention and emotion processing (95% Confidence Interval Lower bound of 66.55 and 82.57, respectively, χ2 (7) = 32.8, *p* < 0.001). LH patients showed a median performance on RTNT, with significantly lower scores for selective attention and working memory (95% Confidence Interval Lower bound of 51.12, χ2 (5) = 20.31 *p* < 0.001) and minimum scores for the same task and for the Stroop test (χ2 (5) = 17.86, *p* < 0.005). The delta for accuracy between the first and the last RTNT run was not statistically significant (RH patients: χ2 (7) = 10.49, *p* > 0.05, n.s.; LH patients: χ2 (5) = 3.35, *p* > 0.05, n.s.). Mean extent of resection was 95.33% ± 9.72 for the RH group and 94.64% ± 6.74 for the LH group. Patients showed good performance post- vs. pre-surgery. The greater difference in the number of LH patients scoring within the normal range was found for the symbol-digit modality test (83.3% to 62%), Stroop test (100% to 77%) and short-term memory (84.61% to 72.72%) and working memory (92.3% to 63.63%). For RH patients, the main changes were observed on the clock drawing test (100% to 77.7%) and cognitive estimation (100% to 72.7%). Conclusions: Frontal RTNT offers continuous and reliable feedback on the patients’ cognitive status during resection in frontal areas.

## 1. Introduction

Our adaptation to the environment is not limited to the abilities to perceive, speak, read, memorize, and move. Daily life activities require us to continually modulate these cognitive skills to suit changing needs, goals, and objectives. Executive functions [1] are defined as “the ability that enables a person to establish new patterns of behavior and ways of thinking and to have introspection about them“. This definition implies that executive functions involve a set of cognitive processes, namely planning, use of strategies, problem solving, abstraction, inhibition, switching, memory, attention allocation and maintenance, judging, estimation, impulse control, emotional control, and social cognition [2,3]. Because of the multi-component architecture, it is not easy to identify a task that can provide evidence of a selective deficit in executive functions [4]. This raises a methodological problem when designing methods to monitor executive functions in the context of awake surgery of frontal lobe lesions.

First, the set of cognitive processes that are part of executive functions have been rarely assessed in awake surgery [5], as they represent extra-language related functions. This is a limitation, as impairments in executive functions affect patients’ quality of life [6]. Few studies report the experience of different neurosurgical teams in testing cognitive processes; for a review, see [7]. According to the literature, the Stroop test is generally used intraoperatively, namely during Direct Electrical Stimulation (DES) of frontal areas. The Stroop test measures the inhibition of cognitive interference during automatic processing, which is one component of executive functions. Nine out of 232 studies considered in the review on DES tasks tested functions other than language or motor abilities, such as executive functions, face recognition, musical skills, and finger gnosis [7]. For instance, interference effects were reported [8] while nine patients with frontal glioma performed the Stroop task during DES to the left anterior cingulate cortex. Other authors [9] used the Stroop task in 27 patients and reported errors while DES was applied to white matter sites below the left inferior and middle frontal gyri, anterior to the insula and over the putamen. A different approach was used [10] for three patients with diffuse low-grade glioma in the left frontal cortex [10]: during electrocorticography, an increasing-difficulty executive function–related task was administered to map executive function complementarily to DES to guide resection.

Second, monitoring of executive functions in the frontal lobe of the non-dominant hemisphere is even less frequent; there is limited experience and few neurosurgical teams use awake surgery in this case [11,12]. However, there are reports of post-surgery neuropsychological deficits after right frontal resections [13,14,15,16]. The Stroop test was used [14] during DES in 34 patients with frontal right hemisphere glioma, and positive sites were found in the inferior fronto-striatal tracts and anterior thalamic radiation. In another report, the Stroop test and short-term memory and working-memory tasks were used [17] along with DES during right frontal lobe resection in a patient with LGG, specifically targeting the frontal aslant tract. DES interfered with working memory and inhibitory functions.

Lastly, the DES methodological approach raises an additional problem in executive function testing during awake surgery. Executive functions are organized in large-scale brain networks, e.g., the fronto-parietal control network, the default mode network, and the dorsal and ventral attention networks [18]. DES aims at interfering with a processing occurring in one or more of these networks; however, this occurs in a focal manner [19,20]. Higher cognitive function–related areas will most probably show no response to DES. In addition, given time constraints during surgical procedures, it is not possible to test all of these aspects of executive functions.

In 2016, an intraoperative monitoring technique called Real-Time Neuropsychological Testing (RTNT, [20]) was validated on a series of 92 patients. RTNT is a testing approach applied throughout the resection phase—and thus is not performed during DES— with repeated tasks selected for the area under resection. When applying this method, resection is guided by continuous evaluation of cognitive functions. It was shown [20] that at 1 week post-surgery, scores were very similar to those detected by RTNT, confirming the validity of the online technique as a predictive tool. At follow-up, the majority of cognitive scores were still >70%, which shows a decrease of <30. Scores thus provide the neurosurgeon with an indication of the effects of resection on the patient’s cognitive performance. A 70% decrease in the patient’s performance vs. baseline is a red flag alerting to possible temporary or permanent discontinuation of the resection. Deterioration occurs gradually; the neuropsychologist assesses whether the decrease is due to the resection or other variables.

We developed a protocol consisting of nine tests for LH frontal resections and eight tests for RH frontal resections, to be administered during RTNT. We chose neuropsychological tests that are often used in pre- and post-surgery. Following the literature on frontal lobe-related functions [1,8,9,17,21,22], we selected tests assessing attention, short-term memory, working memory, and verbal monitoring, as well as abstract language processing [23] and emotion processing [24,25].

Our hypothesis was that our ability to detect any deterioration of cognitive ability could be increased by using RTNT. During DES, tests are administered while the excision is paused. Between two consecutive DES—while resection continues—there is an information gap when no feedback on cognition is collected. The RTNT recalls the IONM concept. It is a monitoring technique that is applied continuously during resection. In this way, there is no information gap between two consecutive surgical phases. DES is the gold standard and should be used. We argue that RTNT is complementary to DES and could be used together with DES to increase the amount of information available to the surgeon. So, the patient’s cognitive status can be better monitored, with executive functions being multi-component (see above). The RTNT includes tests assessing the use of strategies (fluency test), abstraction (metaphor comprehension test), inhibition (Stroop test), attention allocation and maintenance (attentional matrices), emotional processing (IAPS), memory (short-term memory and working memory), social cognition (theory of mind test), and selective attention (Symbol Digit Modalities Test).

Below we describe the protocols and intra-operative performance of a group of 29 patients who underwent surgery for frontal brain tumors.

## 2. Materials and Methods

### 2.1. Participants

A consecutive series of 29 (15F, 14M) adult patients (age ≥ 18 years) different from those included in [20] was retrospectively reviewed. Exclusion criteria included: (i) patients with frontal lesions invading the fronto-insular area, (ii) patients with lesions involving Broca’s area, and (iii) patients with premotor lesions, since the corresponding RTNT protocols were already presented in previous contributions [26,27]. Inclusion criteria were: admission to a neurosurgical ward between 2011 and 2021 for a left- or right-hemisphere tumor involving the prefrontal areas of the left (LH) and the right (RH) hemisphere, and confirmed diagnosis based on the 2016 World Health Organization classification criteria [28]; being native Italian speakers; having normal or corrected-to-normal vision; no history of psychiatric disease or drug abuse, developmental language problems, or learning disabilities and no family history for such disabilities.

RH patients had a mean age of 44.5 years (SD = 13.2 years) and mean education of 14.74 years (SD = 3.8 years). LH patients had a mean age of 40.5 years (SD = 13 years) and mean education of 13.4 years (SD = 3.3 years). They had high-grade glioma (n = 4 LH and n = 5 RH) and low-grade glioma (n = 10 RH and 10 LH). All patients received a neuropsychological battery and an MRI study pre-operatively. All LH patients (n = 15) were right-handed. In the RH group (n = 14), 1 patient was left-handed, 3 patients were ambidextrous, and the others were right-handed. The study was approved by the Ethics Committee (0004890/P/GEN/ARCS, ID 4202) and carried out in accordance with the 2013 Fortaleza version of the Helsinki Declaration and subsequent amendments. As the study was retrospective, written consent to participate in the study was not applicable. Written informed consent was obtained for surgery.

### 2.2. Pre- and Post-Surgery Neuropsychological Assessment

Patients were tested pre- and post-surgery (1 week after resection). Some tests were administered to both LH and RH patients, while others were specific for LH- and RH- related functions and were thus administered to only LH or RH patients. Table 1 lists all tests used for LH and RH patients. To determine whether patients’ scores were within or below the normal range, their performance was corrected for age, education, and sex according to published norms. Scores were compared with published cut-off values.

### 2.3. Real-Time Neuropsychological Testing (RTNT) in Prefrontal Areas

There is a substantial difference between RTNT and DES testing. RTNT is not performed during DES nor during intraoperative mapping; it is administered independently, while the surgeon proceeds with the resection phase (see introduction). RTNT is a sequence of several neuropsychological tasks that are continuously alternated. The task sequence followed a fixed order (e.g., 10 items of each task).

In our study, as resection progressed, sequences (or RTNT runs) of neuropsychological tests were thus continuously repeated (see Table 2). The number of times tests were administered depended on how long the surgery lasted. For instance, the following was applied for RH: 1 item from the ST, digit span forward, digit span backward, 14 items from the SDMT, 10 items from the MC, 10 items from LT, 10 items from IAPS, and 10 items from AT. The sequence of tasks (1st RTNT run) was repeated (presenting a different stimulus sublist for each sequence) until resection ended. Item responses were simple and clear, without multiple levels of response. The number of correct responses was counted. Data from RTNT were interpreted based on the patient’s correct responses ranging from 0–100%, compared to their pre-surgery level (RTNT uses percentages, 100% being optimal brain function). All 29 patients underwent RTNT during resection.

### 2.4. MRI Structural Data

Data were obtained by retrospectively analyzing structural images routinely acquired pre-surgery. A 3-T Philips Achieva whole-body scanner was used to acquire structural data using a SENSE-Head-8 channel head coil. Volumes of interest of the patients’ lesions were drawn on their T1 MRI scans using MRIcron software (https://www.nitrc.org/projects/mricron). The volumes of interest were normalized to the Montreal Neurological Institute space using the “Clinical Toolbox” (https://www.nitrc.org/projects/clinicaltbx/) for SPM8 (https://www.fil.ion.ucl.ac.uk/spm/).

### 2.5. Statistical Analysis

Normal distribution of RTNT data was assessed using the Shapiro–Wilk test. Non-parametric statistics were applied using either the Kruskal–Wallis H test to evaluate a statistically significant difference—if any—in the patients’ median scores (and minimum scores) between tasks or the Mann–Whitney U test, when appropriate. Significance was set at *p* < 0.05. Data were analyzed using SPSS 21.0 (SPSS, Inc., Chicago, IL, USA).

## 3. Results

### 3.1. MRI Analysis

**Figure 1:**
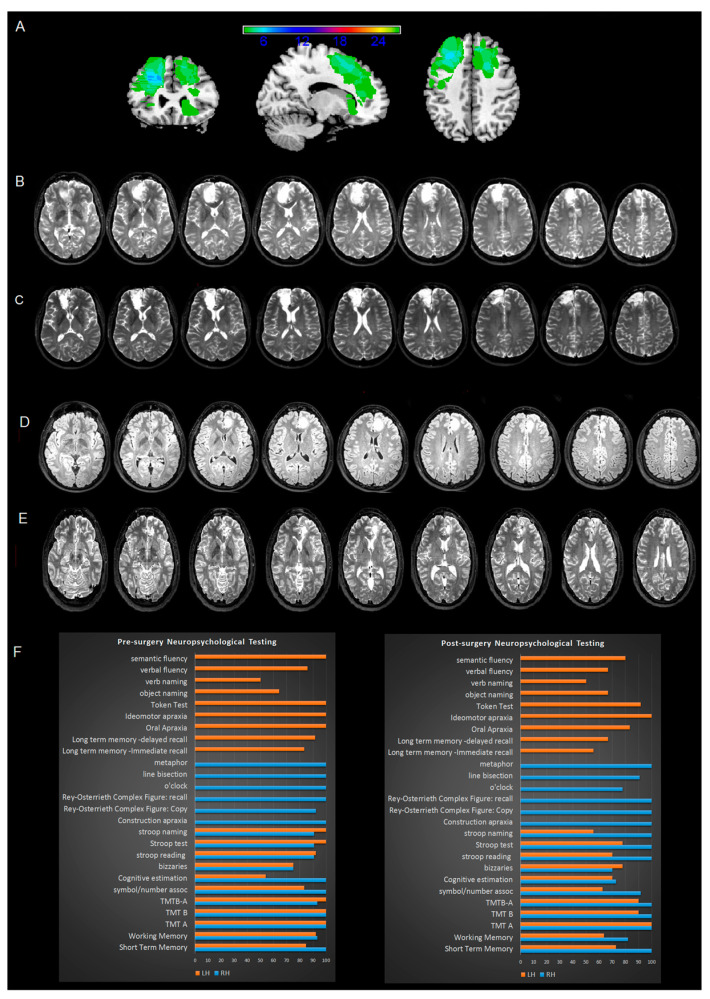
Patients’ lesion volumes of interest (VOIs) overlay (**A**) in addition to pre-surgery (**B**,**D**) and post-surgery (**C**,**E**) T2 MRI images of two representative patients. In (**F**), the % of patients’ scoring within the normal range during the pre- and post-surgery neuropsychological performance is shown.

The maximum lesion overlay of patients’ lesion Volume of Interest (VOI) involved the superior and middle frontal gyrus, and at subcortical level, the anterior and superior corona radiata of LH and RH (Figure 1A, Table 3).

### 3.2. Pre-Surgery Neuropsychological Assessment

The patients’ neuropsychological profile confirmed that they were good candidates for awake surgery (see Figure 1F and Supplementary Table S1).

### 3.3. DES Mapping

All patients underwent awake surgery. After dura opening, 4 motor mappings and 11 language mappings (counting and naming) were performed, and RTNT was directly started in 7 patients. Negative mapping was found in 13 patients (6 LH and 7 RH), a positive motor site was found in 2 LH and 1 RH patients (mainly face, or mouth area), and a positive language-related site (mainly speech arrest) was found in 2 RH and 2 LH patients.

Intra-surgery, DES, and RTNT were intermingled by performing 8 motor mappings and 4 language mappings (counting and naming), and 17 patients performed only RTNT. Negative mapping was found in 5 patients (2 LH and 3 RH), a positive motor site was found in 2 LH and 3 RH patients (mainly face or mouth area), and a positive language-related site (mainly speech arrest) was found in 1 LH patient.

### 3.4. RTNT Results

The Shapiro–Wilk test showed a non-normal distribution, thus non-parametric statistics were applied. The first RTNT run also served as baseline (see Table 4 for the median value during the first RTNT run).

### 3.5. Right Frontal RTNT

**Figure 2:**
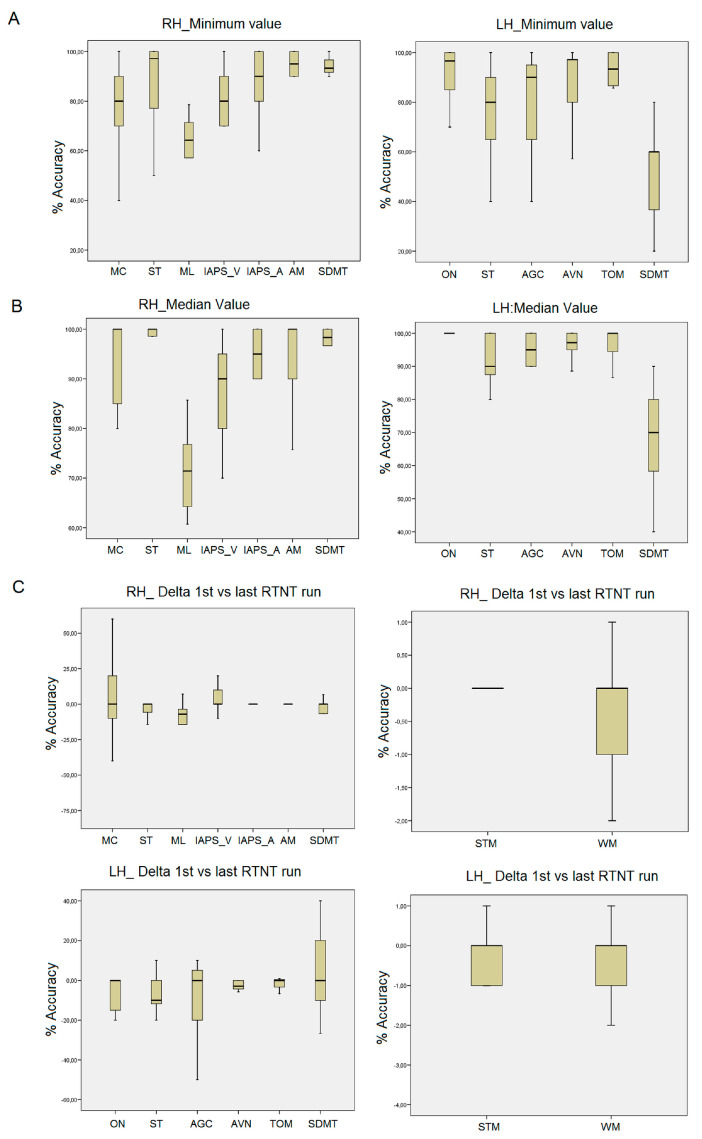
Patients’ RTNT performance during resection. The minimum value (**A**), median value (**B**), and delta (1st vs. last RTNT run, (**C**)) for RH and LH groups are shown. MC = Metaphor comprehension; ST = Stroop test; ML = Milner landmark test; IAPS_V and IAPS_A = international affective picture stimuli, valence and arousal; AM = attentive matrices; SDMT = symbol digit modality test; STM = short-term memory; WM = working memory; ON = object naming; AGC = auditory grammar comprehension; VF = verbal fluency; AVN = action verb naming; TOM = theory of mind.

Overall, the median values (across RTNT runs) for ML and IAAPS_V showed the greatest variation (95% Confidence Interval Lower bound 66.55 and 82.57, respectively; see Table 4). A Kruskal–Wallis H test showed a statistically significant difference in the patients’ median values between tasks, χ2 (7) = 32.8, *p* < 0.001 (see Figure 2). The ML median score significantly differed from the other tasks (vs. MC, U = −3.94, *p* < 0.001; vs. ST, U = −4.3, *p* < 0.001; vs. IAPS_V and IAPS_A, U = −3.4, *p* < 0.001 and U = −3.36, *p* < 0.001; vs. AM, U = −3.71, *p* < 0.001 and vs. SDMT, U = −3.58, *p* < 0.001) and IAPS_V significantly differed from ST (U = −2.78, *p* < 0.005) and SDMT (U = −2.076, *p* < 0.05).

The minimum scores for ML and MC were the ones that varied most (95% Confidence Interval Lower bound 53.67 and 66.89, respectively). A Kruskal–Wallis H test showed a statistically significant difference in the patients’ minimum scores between tasks, χ2 (7) = 24.73, *p* < 0.001 (see Figure 2). The ML minimum value significantly differed from the others (vs. ST, U = −2.61, *p* < 0.001; vs. IAPS_V and IAPS_A, U = −3.37, *p* < 0.001 and U = −3.11, *p* < 0.001; vs. AM, U = −3.21, *p* < 0.001 and vs. SDMT, U = −3.66, *p* < 0.001), and MC significantly differed from AM (U = −2.01, *p* < 0.05).

The delta for accuracy between the first and the last RTNT run was not statistically significant, χ2 (7) = 10.49, *p* > 0.05, n.s. (see Figure 2).

### 3.6. Left Frontal RTNT

Overall, the median values (across RTNT runs) for the SDMT showed the greatest variation (95% Confidence Interval Lower bound 51.12; see Table 4). A Kruskal–Wallis H test showed a statistically significant difference in the patients’ median values between tasks, χ2 (5) = 20.31, *p* < 0.001 (see Figure 2). The SDMT median score significantly differed from the other tasks (vs. ON, U = −3.707, *p* < 0.001; vs. ST, U = −2.67, *p* < 0.001; vs. AGC, U = −2.62, *p* < 0.001; vs. AVN, U = −3.42, *p* < 0.001 and vs. TOM, U = −3.18, *p* < 0.001). The minimum scores for SDMT and ST were the ones that varied most (95% Confidence Interval Lower bound 31.68 and 65.31, respectively). A Kruskal–Wallis H test showed a statistically significant difference in the patients’ minimum scores between tasks, χ2 (5) = 17.86, *p* < 0.005 (see Figure 2). The SDMT minimum value significantly differed from the others (vs. ON, U = −3.44, *p* < 0.001; vs. ST, U = −2.48, *p* < 0.05; vs. AGC, U = −2.58, *p* < 0.005; vs. AVN, U = −2.69, *p* < 0.005 and vs. TOM, U = −3.27, *p* < 0.001), and ST significantly differed from ON (U = −2.055, *p* < 0.05). The delta for accuracy between the first RTNT and last RTNT run was not statistically significant, χ2 (5) = 3.35, *p* > 0.05, n.s. (see Figure 2).

VF was analyzed separately, as there is no accuracy; this measure is expressed as the maximum number of words produced following a given criteria in one minute. Overall, the median values (across RTNT runs) for VF (10.45) did not vary most (95% Confidence Interval Lower and Higher bound 7.86 and 13.03, respectively; see Table 4) nor did the minimum scores for VF (95% Confidence Interval Lower and Higher bound 5.57 and 9.02, respectively).

### 3.7. Short-Term Memory and Working Memory

STM and WM measure the patients’ span and were analyzed separately. For the RH group, the median baseline level at the first RTNT run for STM and WM was 5 and 4. These span levels were maintained as equal (median values across RTNT runs: 5 and 4 and minimum scores: 5 and 4), with a significant difference between the two tests (χ2 (1) = −4.27, *p* < 0.001 and χ2 (1) = −4.004, *p* < 0.001, respectively). For the LH group, the median baseline level at the first RTNT run was 6 and 3, respectively. These span levels were maintained as equal for the median values (across RTNT runs: 5.5 and 3), while the minimum scores, especially for STM, decreased by 2 points (4 and 3), with a significant difference between the two tests (χ2 (1) = −3.001, *p* < 0.001 and χ2 (1) = −3.4, *p* < 0.001). Despite this variation in STM, the difference in the patients’ delta scores between tasks was not statistically significant, both for the LH (χ2 (1) = −0.22, *p* > 0.05, n.s.) and the RH (χ2 (1) = −1.32, *p* > 0.05, n.s.) patients (see Table 4 and Figure 2).

### 3.8. Narrative Language

As no psycholinguistic analysis of speech was performed, figure descriptions were used from a qualitative point of view. The number of patients who did not provide an adequate description were 4/15 RH patients and 5/14 LH patients, and the reasons were poor productivity (3 cases), failure in understanding the story (3 cases), confabulation (2 cases), and intrusions (1 case).

### 3.9. Other Intraoperative Qualitative Observations

As patients are awake and collaborating throughout the resection phase, the RTNT allows the detection of behavioral responses (if they occur): crying (1 RH patient), stomach/nausea sensations (1 RH patient and 1 LH patient), complaining about pain (1 RH patient and 2 LH patients), and falling asleep (1 RH and 2 LH patients).

### 3.10. Extent of Resection

The mean extent of resection was 95.33% ± 9.72 for the RH group and 94.64% ± 6.74 for the LH group.

### 3.11. Post-Surgery Neuropsychological Assessment

Overall, patients performed well at post-surgery testing. As compared to their pre-surgery scoring, there were slight improvements and slight decrements (see Figure 1F and Supplementary Table S2) in the number of patients scoring within the normal range. The greater differences were found for the LH patients on SDMT (83.3% to 62%, 1 LGG pre-surgery, 3 LGG post-surgery), ST (100% to 77%, 0 patients pre-surgery, 2 LGG post-surgery) VF (85% to 66%, 2LGG pre-surgery to 4 LGG post-surgery) and STM (84.61% to 72.72%, 1 LGG pre-surgery, 2 LGG and 1 HGG post-surgery) and WM (92.3% to 63.63%, 2 LGG pre-surgery, 3 LGG and 1 HGG post-surgery). For the RH patients, the greater differences were found on the Clock drawing test (100% to 77.7%, 0 patients pre-surgery, 1 HGG and 1 LGG post-surgery) and cognitive estimation (100% to 72.7%, 0 patients pre-surgery, 2 HGG and 1 LGG post-surgery).

## 4. Discussion

Areas of the frontal lobes preside over cognitive functions whose functioning is based on a neural network. Resections occurred in the superior and middle frontal gyrus and the white matter underneath. RTNT allowed monitoring performance in many more cognitive abilities related to the frontal lobe. The two RTNT protocols for the LH and the RH included tasks that were sensible to the consequences of surgery and were worth monitoring. In particular, for the RH patients, the median performance on a visuo-spatial attention test (ML) and emotion induction test (IAPS) and minimum values on the MC test showed the greater variation. For the LH patients, this was seen on a task monitoring selective attention and working memory (SDMT), the ST, STM, and WM. Importantly, the delta for accuracy between the first and the last RTNT run was not statistically significant, meaning that the cognitive status was maintained, despite the slight changes reported above. This is exactly what the RTNT is meant to measure: monitoring functions and detecting early changes [20]. Interestingly, there is a correspondence between the tasks that become more sensitive during resection and post-surgery evaluation. As compared to pre-surgery, post-surgery evaluation showed that the larger changes (namely, patients within the normal range post-surgery) for the LH patients were found for SDMT (83.3% to 62%), ST (100% to 77%), STM (84.61% to 72.72%), and WM (92.3% to 63.63%); for the RH patients, the larger changes were found instead for the Clock drawing test (100% to 77.7%) and cognitive estimation (100% to 72.7%). The average resection in these brain areas was 95.76% ± 6.17%. The neuropsychological literature shows that maintenance of executive functions is relevant to ascertain the patients’ quality of life [6,7,8,9,10,11,12,13,14].

Looking at previous studies, few of them report the experience of different neurosurgical teams in testing one aspect of executive functions only (for a review, see [7]). Our results on LH patients are consistent with studies on brain tumor patients reporting SDMT deficits in frontal glioma patients [46,47]. Similarly, the ST is the one most frequently used tasks during DES studies on frontal lobe patients [8,9,17]. Lastly, regarding STM and WM, the role of frontal areas in encoding processes does not appear to be specific to memory. The frontal lobes are supposed to manage control processes that allow selection of relevant information, inhibition of irrelevant information, or management of working memory, in accordance with the individual’s goals and motivational states [1]. In addition, our results on RH patients are consistent with some previous studies. It has been shown that resection of a part of the medial superior and middle frontal gyri was correlated to low visuospatial cognitive accuracy [13,16]. Lastly, post-surgery, resection in the right frontal lobe can cause impairments in MC [16] as well as impairments in emotion processing [48]; for a review, see [49]. In a few neuropsychological follow-up studies, cognitive deficits have emerged following resection of the frontal lobe [13,14,15,16]. It follows that it is worth monitoring cognition during resection in the frontal cortex. Narrative language deserves further testing and analyses. It allowed us to detect poor initiative/ productivity, an inability to understand the meaning of the cartoon, confabulatory speech, and intrusions. This is consistent with the role of the frontal cortex in narrative language [50,51,52]. There is a debate about how to test complex cognitive functions intra-surgery [19]. The frontal RTNT protocol allows the usage in intra-operative neuropsychological monitoring of more tasks. The RTNT enables the use of many more tests than DES (owing to time constraints). In particular, visuo-spatial planning and cognitive estimation can be adapted to the operatory room and included in monitoring (as they are among the tasks that showed greater change post-surgery). This view supports the use of mapping and monitoring even with right hemisphere resections [12].

## 5. Conclusions

Our data show that the RTNT protocols assisting the surgeon during frontal resection include tasks that are feasible and provide significant data with respect to the patients’ post-surgery neuropsychological status.

Monitoring of executive functions in the frontal lobe is less frequently used as compared to language and motor mapping, and few neurosurgical teams use awake surgery in this case [8,9,10,11,12,13,14]. However, evidence for post-surgery neuropsychological deficits after frontal resections has been reported [13,16,46]. We monitored the aspects of executive functions using the RTNT approach and a number of tests, without prolonging the length of surgery. This was made possible by the fact that the tests are administered while the surgeon carries out the resection. RTNT taught us that achieving resection in the shortest time is of the utmost importance. Since the surgeon receives more feedback on the patients’ cognitive status and may thus feel more confident, resection time is shorter [20].

## Figures and Tables

**Table 1:** Pre- and post-surgery neuropsychological assessment.

Function	Test	LH/RH Protocol
Handedness	Oldfield [29]	LH and RH
Abstract reasoning	Raven Matrices [30]	LH and RH
Psychomotor speed and selective attention, attention shifting and cognitive flexibility	Trail Making Tests A and B [21]	LH and RH
Reasoning, development and application of appropriate strategies, and response plausibility	Cognitive Estimations [22]	LH and RH
Inhibition of cognitive interference that occurs during automatic processing	Stroop Test [23]	LH and RH
Motor speed, attention, and visuo-perceptual functions	Digit Symbol Substitution Test [24]	LH and RH
Short-term memory	Digit span forward [25]	LH and RH
Working memory	Digit span backward [25]	LH and RH
Selection of words meeting certain constraints and repetition avoidance, both based on executive control processes	Verbal and Semantic fluency [31]	LH
Visuo-spatial planning	O’Clock Test [32]	RH
Analytical thinking in the verbal domain	Comprehension of metaphors and idioms [33]	RH
Object and verb naming	Battery for the analysis of language disorders [34]	LH
Imitation	Ideomotor apraxia [35] and oro-facial [36]	LH
Verbal comprehension	Token Test [37]	LH
Visuo-spatial processing	Figure copy [38]	RH
Visuo-spatial attention	Line Bisection [39]	RH
Visuo-spatial memory	Rey-Osterrieth Complex Figure Test [40]	RH

**Table 2:** RTNT protocols for LH and RH frontal resections.

RTNT Test	LH/RH Protocol		Assessed Abilities	Tests Identically Used Intraoperatively vs. Pre- and Post-Surgery
Object Naming task (ON) [34]	LH	The patient is presented with a series of black and white line drawings and asked to name the corresponding object associated with it.	lexical access	V
Stroop test (ST) [23]	LH and RH	The patient is asked to read the names of colors, name the colors of squares, and say the color of the ink with which the name of the words is written while inhibiting reading of the name itself (e.g., say “red” in response to the word “green” written in red ink).	selective attention, inhibition of irrelevant information	V
Auditory Grammar Comprehension (AGC) [34]	LH	The neuropsychologist reads a sentence, and the patient is required to say which image, choosing between two options, represents the meaning of the sentence. Sentences are reversible items, e.g., active or passive forms with a transitive verb and two nouns, e.g., the horse is chasing the girls (#1: a picture depicts the horse chasing the girls, #2: the girls are chasing the horse).	ability to understand semantically reversible sentences (active or passive) by choosing between two images, divided attention	Indirectly tested by analyzing the Token test performance
Short-term memory (STM) [25]	LH and RH	The patients is asked to repeat each digit sequence in the same order as it is read.	verbal short term memory and attention	V
Working memory (WM) [25]	LH and RH	The patient is asked to repeat each digit sequence by reversing its order.	working memory and attention	V
Verbal fluency (VF) [31]	LH	The patient is asked to produce as many words as she/he can in one minute by maintaining a given criteria (the first letter is given by the neuropsychologist). Each RTNT run has a different letter and lasts one minute.	lexical phonologically access speed and verbal monitoring	V
Action Verb Naming (AVN) [34]	LH	Action verb naming was monitored using the oral verb naming task. Patients were presented with a series of black and white line drawings and asked to name the corresponding verb associated with it.	lexical access	V
Theory Of Mind (TOM) [41]	LH	Short stories with one character are verbally presented. At the end of each story, the patient is asked to say which emotion the character is feeling, e.g., “Maria has to make a speech at work. She is standing in the room in front of everyone and cannot remember a word of what she has to say. Everyone is staring at her. How will Maria feel in this situation?”. Each RTNT run includes 5 items.	social cognition, emotion processing	Not tested
Narrative language (NL) [42]	LH and RH	Black and white pictures depicting a short story are shown. Each is divided into 4 vignettes following a time line. The patient is asked to tell the story depicted. One picture is presented for each RTNT run.	lexical access, verbal monitoring	Indirectly tested by analyzing the patient’s speech during the clinical interview
Symbol Digit Modalities Test (SDMT) [43]	LH and RH	The patient is asked to verbally substitute a symbol with a corresponding digit. A grid reporting symbol–digit correspondences is shown on the top of the image. A row of 15 symbols is shown below, and the patient is asked to say the corresponding number (digits 1 to 9).	selective attention, working memory processing speed	V
Metaphor comprehension (MC) [33]	RH	The patient reads a metaphor and has to explain its meaning in his/her own words (literally). Each RTNT run includes 5 metaphors.	abstract language	V
Milner Landmark Test (LT) [44]	RH	Lines, which are divided into two segments of different length, colored in red and black are shown in the center of the screen. The patient is required to decide which segment (red or black) is the longest (or alternatively the shortest), according to the instruction.	visual attention	Indirectly tested by analyzing the visuospatial attention tasks
International Affective Picture System (IAPS) [45]	RH	Emotionally charged (negatively or positively) images or neutrals are presented. For each image, the patient is asked to indicate on a Likert scale (from 1 to 9) how he/she feels when looking at that image. For each image, there are 2 answers: pleasure (happy vs. unhappy scale) and arousal (excited vs. calm dimension).	emotional processing, monitoring internal emotional states and arousal	Not tested
Attentional Matrices (AT) [38]	RH	Matrices, composed by a series of numbers and a given target number, are presented. The patient is asked to report how many times the target number(s) appears within the different rows. To facilitate visual search, the test was adapted so that both the target number and the row are underlined in red. Each RTNT run includes 1 attentional matrix.	selective attention, working memory,	Indirectly tested by analyzing performance on the TMT, Stroop test, and SDMT

**Table 3:** Spatial coordinates, according to the Montreal Neurological Institute space system, of the brain areas listed in the first column, with the worst damage, as evidenced by the percentage lesion overlay indicating the percentage of patients with a lesion maximally localized in the listed area.

Area	% N > 0	MaxX	MaxY	MaxZ	%
Frontal_Sup_RH	82	29	25	33	50
Frontal_Mid_RH	93	30	25	33	50
Frontal_Sup_LH	74	−13	34	38	40
Frontal_Mid_LH	55	−26	16	43	40
Superior_corona_radiata RH	68	23	12	24	42.85
Anterior_corona_radiata RH	94	25	13	24	42.85
Superior_corona_radiata LH	38	−24	13	28	26.66
Anterior_corona_radiata LH	99	−17	34	10	26.66

**Table 4:** Patients’ RTNT performance during resection.

RTNT_Task	Median Value during the First RTNT Run (Baseline)	Mean	Sd	Median	95% Confidence INTERVAL Lower	95% Confidence INTERVAL Upper
RH patients						
MC	90	93.33	8.87	100	87.69	98.97
ST	100	97.44	5.66	100	94.17	100.71
ML	78.57	71.74	7.73	71.42	66.55	76.94
IAPS_V	90	88.26	9.43	90	82.57	93.96
IAPS_A	100	91.73	11.15	95	84.99	98.46
AM	100	94.98	8.13	100	88.75	100.38
SDMT	100	96.66	5.63	98.66	91.95	101.37
STM	5	5.35	0.66	5	4.97	5.73
WM	4	3.8	0.60	4	3.33	4.27
LH patients						
ON	100	97.91	4.98	100	85.4	101.08
ST	100	90.90	11.13	90	65.31	98.39
AGC	90	91.36	12.46	95	67.32	99.73
STM	6	4.45	1.29	4	3.58	5.32
WM	3	2.7	0.67	3	2.21	3.18
VF	12	10.45	3.61	9.5	7.86	13.03
AVN	100	96.62	3.95	97.14	73.93	99.27
TOM	90	96.95	4.98	10	87.92	101.12
SDMT	100	68.09	18.34	70	31.68	85.06

MC = Metaphor comprehension; ST = Stroop test; ML = Milner landmark test; IAPS_V and IAPS_A = international affective picture stimuli, valence and arousal; AM = attentive matrices; SDMT = symbol digit modality test; STM = short-term memory; WM = working memory; ON = object naming; AGC = auditory grammar comprehension; VF = verbal fluency; AVN = action verb naming; TOM = theory of mind.

## Data Availability

The datasets analyzed for this study will be made available by the authors upon request.

## Supplementary Materials

The following supporting information can be downloaded at: https://www.mdpi.com/article/10.3390/curroncol30020156/s1, Table S1: Pre-surgery neuropsychological assessment; Table S2: Patients’ post-surgery neuropsychological scores.
