# Supplementary material for: Continuous Real-Time Neuropsychological Testing during Resection Phase in Left and Right Prefrontal Brain Tumors
Source: Curr Oncol. 2023 Feb 6;30(2):2007–20. doi: 10.3390/curroncol30020156 (PMC9955514; doi:10.3390/curroncol30020156)
Supplement: Supplementary file 1 [file curroncol-30-00156-s001.zip › curroncol-2069369-supplementary.pdf]

Supplementary Material

Table S1 Patients' pre-surgery neuropsychological scores

P	age	school	RCM	STM	WM	TMT A	TMT B	TMT B-A	s/nass	Cognitive estimation	bizz	stroop reading	Stroop test	stroop naming	Construction apraxia	Copy Rey	Rey Del	o'clock	line bisection	metaphor	MLT - RI	MLT- RD	Oral Apraxia	IMA	Token Test	object naming	verb naming	verbal fluency	semantic fluency
RH#1	49	18	32	5,57	3,77	47	140	93	65	12,2	2				11,25	30,8	18,6	10	8	30,5	na	na	na	na	na	na	na	na	na
RH#2	53	13	26	3,57	2,77	26	76	50	39	16,97	6	54,58	16,89	36,45	12,25	25,8	10,6	10	6	23,25	na	na	na	na	na	na	na	na	na
RH#3	27	17	32	5,27	5,35	44	141	96	47	14,97	2	28,32	8,96	30,7	12,25	33,3	27,5	10	9	29,5	na	na	na	na	na	na	na	na	na
RH#4	27	18	29	6,3	6,68	38	141	102	85	15,97	2	63,32	24,96	42,7	10,25	33,3	11	10	9	21,5	na	na	na	na	na	na	na	na	na
RH#5	45	8	34	4,92	3,68	19	42	23	47	12,97					13	36,4	10,2	10	9	34,75	na	na	na	na	na	na	na	na	na
RH#6	40	18	31	5,43	7,58	28	75	48	63	9,2	1	80,51	18,95	51,18	12,25	33,9	20,7	10	9	24,5	na	na	na	na	na	na	na	na	na
RH#7	72	8	32,5	4,36	4,31	11	-6	-17	43	10,97	1	66,42	16,05	39,79	13	36,2	23,3	9,5	9	26,25	na	na	na	na	na	na	na	na	na
RH#8	63	17	28,5	3,83	4,15	17	207	209	52	17,2	7				12,75	34,4	11,8	10	9	28,5	na	na	na	na	na	na	na	na	na
RH#9	46	13	31	4,67	3,68	35	101	66	58	11,97	1	75,33	45,45	25,89	12,75	35,4	12,8	10	9	29,25	na	na	na	na	na	na	na	na	na
RH#10	40	16	30,5	7,43	7,58	29	91	63	67	9,97	2	75,521	24,95	55,18	12,25	34,2	15,5	10	9	26,5	na	na	na	na	na	na	na	na	na
RH#11	36	18	30,5	5,37	3,5	26	104	72	62	14,2		66,44	36,01	56,92	12,5			10		27,5	na	na	na	na	na	na	na	na	na
RH#12	23	16	30	5,27	5,35	45	128	83	71	14,2	5	65,32	31,96	50,7	12,25	33.5	18	9.5	9	27,5	na	na	na	na	na	na	na	na	na
RH#13	50	8		3,99	3,77	20	56	61	48	16,2	3	74,25	23,7	41,93	11,25	36,7	18,5	10	9	30,75	na	na	na	na	na	na	na	na	na
RH#14	52	16	18,5	4,57	4,77	27	93	66	70	7,2	2	83,58	25,89	46,45	12,75	34,8	10,1	10		32,75	na	na	na	na	na	na	na	na	na
RH#15	45	17	31,5	6,5	4,68	34	92	58	46						12,25			10	9		na	na	na	na	na	na	na	na	na
LH#1	59	11	34	4,92	2,87	11	32	21	39	19,97	6	64,25	24,7	40,93	na	na	na	na	na	na	4,6	5,9	17,75	67	32,25	27	25	20	56
LH#2	30	8	33	4,71	3,79	36	70	44	43	16,97	4	58,47	26,16	46,8	na	na	na	na	na	na	6,3	6	19,75		34,75	28	27	42	59
LH#3	41	17	30	3,47	1,37	43	106	64		15,2	3	8,58			na	na	na	na	na	na	6,6	6,2	19,75	72	32,75	29	27	49	
LH#4	26	16	31	5,3	3,21	36	146	99	61	22,2	7	45,32	31,96	41,7	na	na	na	na	na	na	5	4,3	19,75	69	32,75	24	24	10	27
LH#5	43	18	30,5			32	96	64							na	na	na	na	na	na			19,75	72	31,75	30	28	55	
LH#6	27	13	32,5	6,44	5,42	28	105	76	20	22,97	4	84,09	19,42	57,35	na	na	na	na	na	na	2,8	-0,8	19,75	72	33,5	29	28	28	42
LH#7	55	15	33	3,83	3,79	65	139	75	53	14,97	0	77,67	26,65	48,45	na	na	na	na	na	na	4,7	6,5	19,75	70	33	30	27	37	46
LH#8	39	16	31,5	6,47	4,37	33	91	4	68	13,2	3	69,44	23,01	31,92	na	na	na	na	na	na	6,3	5,9	19,75	72	30,75	29	25	58	48
LH#9	64	13	32,5	7,02	5,97	12	59	47	50	17,2	6	48,81	20,66	47,79	na	na	na	na	na	na			19,75		33,5	27	26	37	53
LH#10	35	13	29	5,55	4,52	25	68	43	47	20,97	1	79,91	25,13	55,34	na	na	na	na	na	na	6,2	5,7	19,75	72	31,5	28	25	37	62
LH#11	35	18	29,5	5,55	4,52	39	103	64	62	11,97	0	66,44	22,01	44,92	na	na	na	na	na	na	7,6	7,2	19,75	72	31,75	29	28	53	54

LH#12	27	11	25	5,65	2,74	41	142	101	55	22,2	0	68,42	26,23	39,55	na	na	na	na	na	na	2,8	4,5	19,75	72	31,75	27	23	14	30
LH#13	27	11	25	4,65	2,74	28	92	64	63	22,2	0	64,42	26,2	40,55	na	na	na	na	na	na	4,2	3,9	19,75	72	33,75	26	24	23	44
LH#14	59	8	36	8,13	4,9	24	91	66	23	16,2		76,65	17,96	42,56	na	na	na	na	na	na	4,6	5,3	19,75	69	33,5	29	25	35	54

P=patient; RH=right heisphere; LH=left hemisphere; school= years of education; RCM=Raven Colored Matrices; STM=Short term memory; WM= working memory; TMT= Trial Making Test; s/n ass= symbol/number association test; biz= bizzaries; del= delayed; MLT=long term memory; RI = immediate recall; RD= delayed recall; IMA=ideomotor apraxia; grey scores denote pathological performance.

Table S2 Patients' post-surgery neuropsychological scores

P	STM	WM	TMT A	TMT B	TMTB-A	s/n ass	Cognitive estimation	bizz	stroop reading	Stroop test	stroop naming	Construction apraxia	Copy Rey	Rey del	o'clock	line bisection	metaphor	MLT - RI	MLT - RD	Oral Apraxia	IMA	Token Test	object naming	verb naming	verbal fluency	semantic fluency
RH#1																		na	na	na	na	na	na	na	na	na
RH#2			33	66	33	40	19,97	7		63,58	16,89	44,45					28,25	na	na	na	na	na	na	na	na	na
RH#3	4,27	4,35	48	121	72	47	12,97		12,25	58,32	18,96	33,7	12,25	33,3	19,5	10	22,5	na	na	na	na	na	na	na	na	na
RH#4	5,3	5,68	46	152	105	78	16,97	2	10,25	78,32	27,96	39,7	10,25	33,3	19	10	23,5	na	na	na	na	na	na	na	na	na
RH#5	5,92	2,68	24	39	15	48	13,97	2	13				13	36,4	8,2	10	35,75	na	na	na	na	na	na	na	na	na
RH#6	4,43	2,58	34	87	54	53	15,2	5	10,25	71,51	25,95	48,18	10,25	32,9	26,7	10	25,5	na	na	na	na	na	na	na	na	na
RH#7	5,36	6,31	6	1	4	43	13,97	2	13	68,42	22,05	37,79	13	38,2	32,3	9,5	29,25	na	na	na	na	na	na	na	na	na
RH#8	4,83	4,15	20	74	73	39	19,2	7	12,75				12,75	35,4	9,8	10	30,5	na	na	na	na	na	na	na	na	na
RH#9	3,67	3,68	39	106	67	41	23,97	4	12,75	65,33	24,45	45,89	12,75	35,4	24,3	10	21,25	na	na	na	na	na	na	na	na	na
RH#10	6,43	5,58	29	91	63	60	13,97	3	12,25	71,51	33,95	53,18	12,25	34,2	8	10	22,5	na	na	na	na	na	na	na	na	na
RH#11	5,37	3,5	33	111	78	58	11,2	4	12,5	74,44	38,01	61,92	12,5			10	27,5	na	na	na	na	na	na	na	na	na
RH#12																		na	na	na	na	na	na	na	na	na
RH#13	3,99	3,77	50	4	3	30	13,2	3	12,25	67,25	16,7	38,93	12,25	34,7	16,5	10	32,75	na	na	na	na	na	na	na	na	na
RH#14	4,57	3,77	30	112	82	57	12,2	1	12,75	83,58	36,89	47,45	12,75	34,8	13,1	5	32,75	na	na	na	na	na	na	na	na	na
RH#15	5,5	5,68	33	82	49	60			11,25				11,25			10		na	na	na	na	na	na	na	na	na
LH#1												na	na	na	na	na	na									
LH#2												na	na	na	na	na	na									
LH#3	3,47	2,37	32	96	65		9,2	2	13,58			na	na	na	na	na	na	6,6	6,2	19,75	72	32,75	30	26	57	
LH#4	5,3	3,21	55	160	104	39	24,2	11	44,32	20,96	26,7	na	na	na	na	na	na	4,1	3,7	19,75	68	29,75	24	24	17	17
LH#5												na	na	na	na	na	na				72	32,75	30	28	54	
LH#6	8,44	5,42	28	96	67	68	19,97	5	71,09	36,42	53,35	na	na	na	na	na	na	1,7	1,4	19,75	72	33,5	30	28	22	39
LH#7	4,83	4,79	29	80	51	49	15,97	2	74,67	17,65	42,45	na	na	na	na	na	na	6,7	2,9	18,75	69	33	29	27	55	42
LH#8	5,47	1,37	39	116	4	63	12,2	3	36,44	11,01	10,92	na	na	na	na	na	na	1,9	1,3	19,75	72	30,75	27	24	7	34
LH#9	6,02	3,97										na	na	na	na	na	na			14,75		34,5	29	27	17	59
LH#10	5,55	2,52	31	130	99	43	21,97	1	41,91	23,13	32,67	na	na	na	na	na	na	6,2	-0,3		72	20,5	24	23	10	27

LH#11	5,55	4,52	40	125	85	41	12,97	0	39,44	16,01	24,92	na	na	na	na	na	na	7,6	7,2	72	31,75	30	28	22	33
LH#12	3,65	1,74	29	113	84	36	17,2	0	58,42	26,2	35,55	na	na	na	na	na	na			69	33,75	25	24	24	44
LH#13	3,65	2,74	26	95	69		17,2	1	53,42	32,2	31,55	na	na	na	na	na	na	3,4	3,1	72	27,75	25	23	21	43
LH#14	6,13	2,9	49	309	229	20	12,2		48,65	4,95	25,56	na	na	na	na	na	na	3,3	3,3	68	28,5	30	25	12	9

P=patient; RH=right hemisphere; LH=left hemisphere; school= years of education; RCM=Raven Colored Matrices; STM=Short term memory; WM= working memory; TMT= Trial Making Test; s/n ass= symbol/number association test; biz= bizzaries; del= delayed; MLT=long term memory; RI = immediate recall; RD= delayed recall; IMA=ideomotor apraxia; grey scores denote pathological performance.
